# Supplementary material for: DRL1, Encoding A NAC Transcription Factor, Is Involved in Leaf Senescence in Grapevine
Source: Int J Mol Sci. 2019 May 31;20(11):2678. doi: 10.3390/ijms20112678 (PMC6600502; doi:10.3390/ijms20112678)
Supplement: Supplementary file 1 [file ijms-20-02678-s001.pdf]

Supplementary Materials

Fig. S1

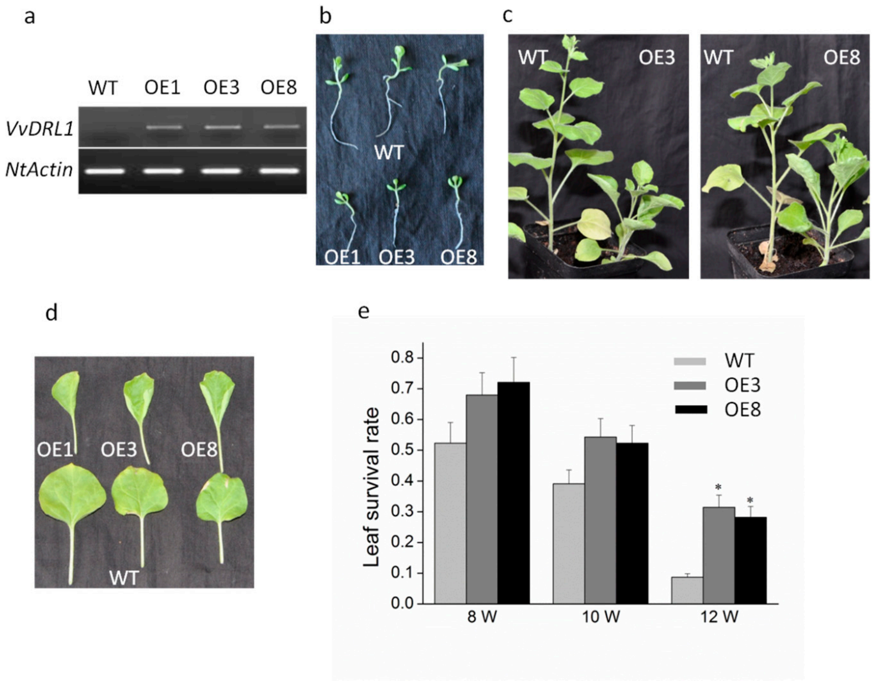

Fig. S1 The phenotype of *DRL1* transgenic tobacco. (a) The expression of *DRL1* in transgenic and WT plants. (b, c) The phenotype of transgenic plants at 1 (b) and 8(c) weeks. (d) The phenotype of leaves from *DRL1* transgenic plants. (e) Leaf survival rate. Bars represent mean ± SD. Significant difference from the WT was confirmed by Tukey's test (* $P < 0.05$)

Fig. S2

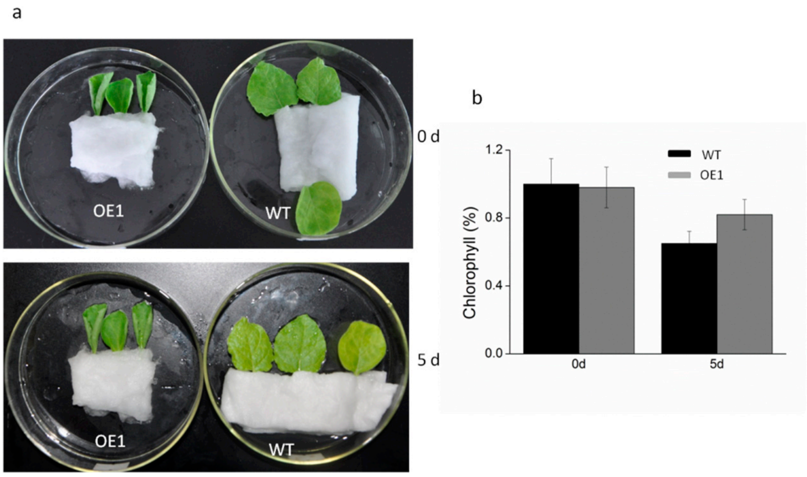

Fig. S2 *DRL1* promotes leaf senescence in tobacco. (a) Detached leaf senescence phenotypes of transgenic tobacco treated with drak for 5d. (b) The determination of chlorophyll concentration. Bars represent mean ± SD with at least three biological replicates.

Fig. S3

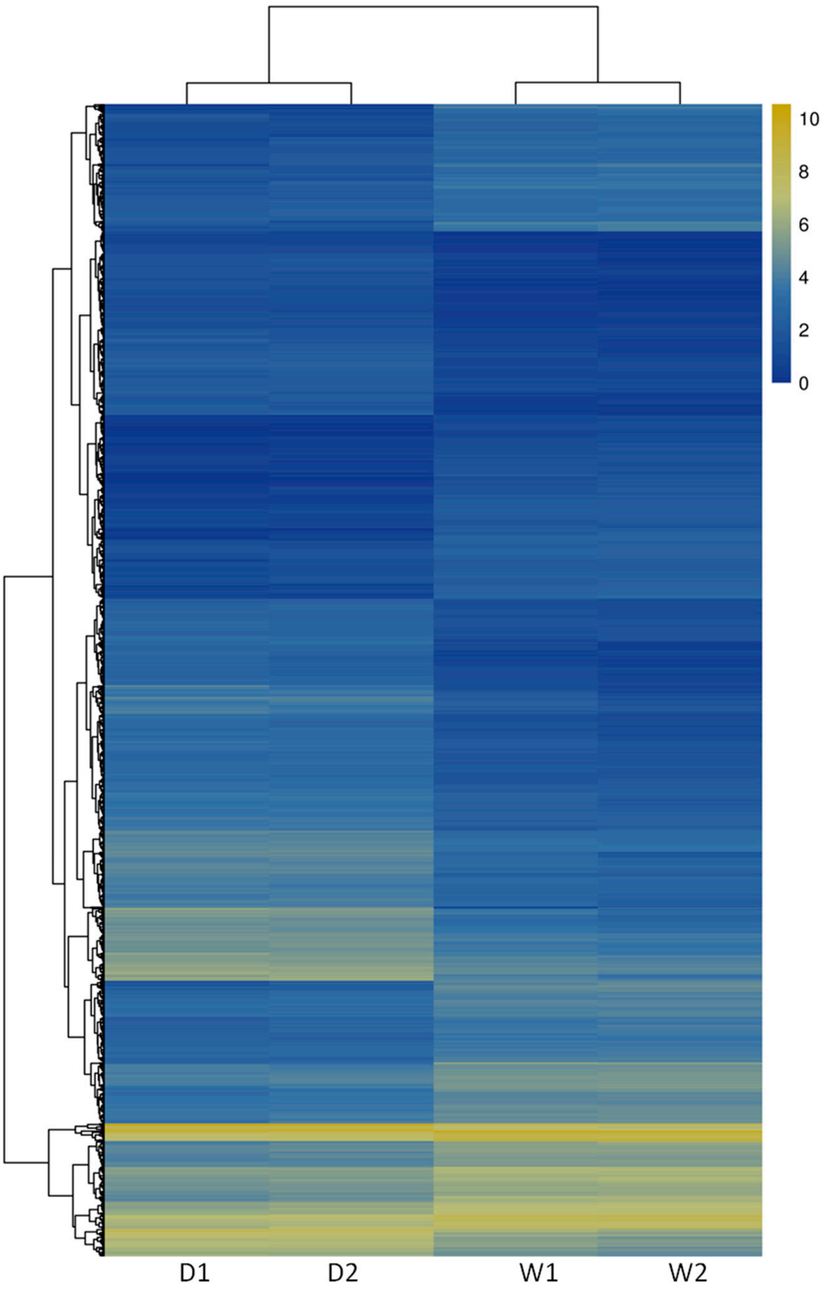

Fig. S3 Heatmap of DEGs in leaves of beased on RNA-seq. The expression levels are quantified as log_2. Sampled D1 and D2 were two biological repicates, W1 and W2 were two biological repicates. Among these expressed transcripts, 25381 transcripts showed no significant changes in their expression level (log2<1), while 1149 transcripts were differentlly expressed in DRL1 transgenic tobacco (log2<1) at false discovery rate (FDR<0.001), which includes (50.8%) up-regulated and (49.2%) down-regulated transcripts.

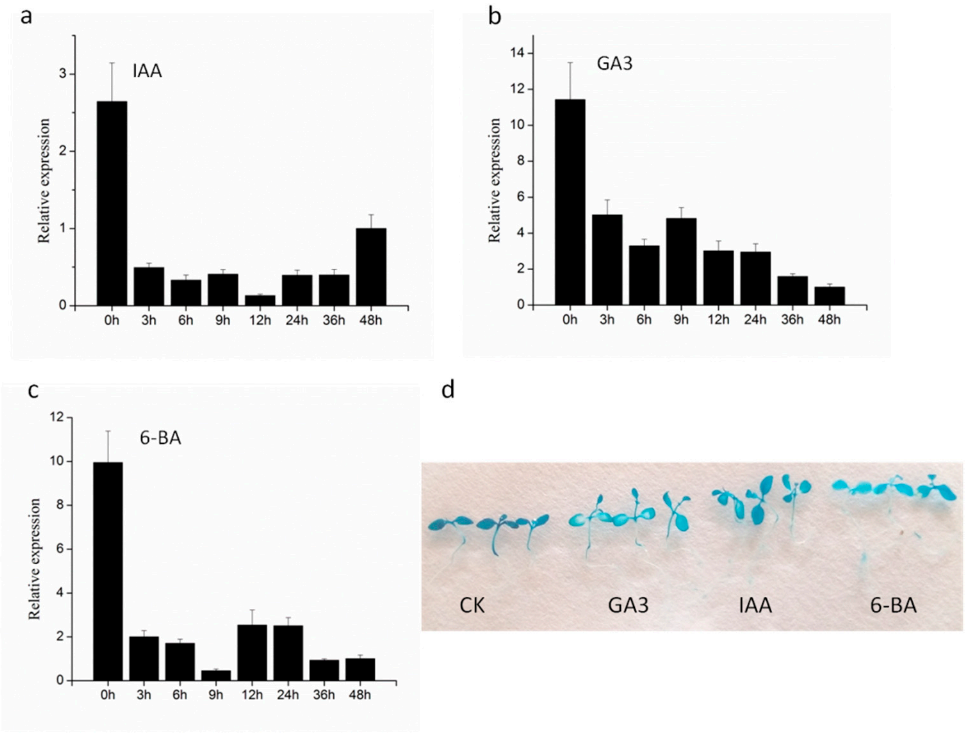

Fig. S4 The expression analysis of *DRL1*. (a) The expression of *DRL1* in grapevine following hormone IAA, GA3 and 6-BA treatment. (b) GUS staining in leaves of P_{DRL1}-GUS transgenic tobacco plants under ABA treatment. Six-day-old tobacco seedlings were treated (n=60).

Table S1 Number of reads sequenced and mapped to the tobacco genome. The transcriptomic sequencing of cDNA generated from both wild type and DRL1 transgenic tobacco leaf samples produced 14 and 14.18 milllion clean reads, respectively, the clean reads of each sample reached 6.78 Gb.

	WT	DRL1	Sum
Clean Reads	23360757	24109158	47469915
Total mapped	17262252	17885486	35147738
Percent of mapped clean reads	73.86%	74.20%	74.03%

Table S2 Unigene annotation. In the whole unigene set, a total of 44265 (46.2%) unigenes were significantly matched to known genes in the public databases of NR, NT, Swiss-Prot, KEGG, COG, and GO. (The total unigene number was 95756)

Annotation Database	Annotated Number	300<=length<1000
COG Annotation	11707	3579
GO Annotation	21807	8150
KEGG Annotation	13205	4990
KOG Annotation	21238	7604

Pfam Annotation	24146	7757
Swissprot Annotation	20973	7382
eggNOG Annotation	37777	14211
nr Annotation	43602	17373
All Annotation	44262	17539

Table S3 DEGs related to hormones.

ID	log2FC	Regulated	Annotation
**IAA related genes**			
c62173	1.678787	up	auxin-induced protein 15A-like
c74273	2.260212	up	auxin-induced protein 15-like
c73867	2.159749	up	auxin-induced protein 16-like
c82466	2.725583	up	auxin-responsive protein IAA16
c75798	2.66065	up	auxin-responsive protein IAA16
c70706	1.782122	up	auxin efflux carrier component 1c
c70976	1.090073	up	auxin efflux carrier component 1c
c48602	1.943798	up	premnaspirodiene oxygenase-like
c62356	1.940161	up	xyloglucan endotransglucosylase/hydrolase protein
c67577	1.033957	up	isoflavone 2'-hydroxylase-like
c77318	1.192652	up	inactive receptor kinase At2g26730
c74045	1.763241	up	cytochrome P450 CYP736A12-like
c66813	2.039682	up	cytochrome P450 CYP72A219-like
c42316	-1.10457	down	indole-3-acetic acid-induced protein ARG2-like
c72652	-1.94887	down	3,9-dihydroxypterocarpan 6A-monooxygenase-like
c75509	-1.08972	down	auxin-induced protein 6B-like
c31554	-1.96442	down	auxin-induced protein 15A-like
c66540	-1.26499	down	auxin-induced protein 6B-like
c81771	-2.02658	down	auxin-responsive protein IAA29-like
c81858	-2.03041	down	auxin-induced protein 15A-like
c81270	-1.15669	down	cytochrome P450 CYP736A12-like
c67966	-3.28212	down	cytochrome P450 86B1-like
c77102	-1.11054	down	cytochrome P450 78A3-like
c81976	-1.98908	down	cytochrome P450 714A1-like
c12113	-3.32038	down	cytochrome P450 93A3-like
c75878	-1.03252	down	cytochrome P450 90A1
c38135	-1.40632	down	cytochrome P450 78A6-like
c76819	-1.38977	down	cytochrome P450 734A1-like
c48010	-1.52565	down	cytochrome P450 704C1-like
c76093	-1.21193	down	cytochrome P450 CYP72A219-like
c81697	-1.88398	down	cytochrome P450 78A3-like
c69215	-1.13064	down	cytochrome P450 78A5-like

**GA related genes**			
c25557	3.545966	up	gibberellin-regulated protein 11-like
c63722	2.632892	up	Gibberellin regulated protein 12
c64833	-3.53212	down	gibberellin 2-beta-dioxygenase-like
c6507	-2.88767	down	gibberellin 2-beta-dioxygenase 2-like
c71394	-1.52512	down	gibberellin 2-beta-dioxygenase 1-like
c77228	-1.33114	down	gibberellin 2-beta-dioxygenase 3e
c23771	-1.05842	down	gibberellin 2-beta-dioxygenase 3
c81696	-1.75872	down	2-oxoglutarate-dependent dioxygenase AOP3-like
**CTK related genes**			
c12570	-2.36607	down	two-component response regulator ARR15
c69612	-1.62696	down	two-component response regulator ARR9-like
c48223	-1.39363	down	two-component response regulator ARR9
c70750	-1.70688	down	cytokinin riboside 5'-monophosphate phosphoribohydrolase LOG1-like isoform X1
c69978	-1.07816	down	cytokinin riboside 5' monophosphate phosphoribohydrolase LOG1-like
**ABA related genes**			
c67931	1.048154	up	xanthoxin dehydrogenase-like
c55983	1.306234	up	abscisic stress-ripening protein 2
c72359	-3.54043	down	zeaxanthin epoxidase, chloroplastic-like
c81325	-1.40141	down	zeaxanthin epoxidase, chloroplastic-like
c77357	-1.13117	down	zeaxanthin epoxidase, chloroplastic
c67782	-3.47452	down	abscisic acid receptor PYL4-like
c72107	-2.00208	down	protein ABSCISIC ACID-INSENSITIVE 5
c44037	-1.28001	down	protein FAF-like, chloroplastic
c75239	-4.64207	down	abscisic acid 8'-hydroxylase 4-like
c64198	-3.11972	down	abscisic acid 8'-hydroxylase 1-like
c25919	-1.4221	down	abscisic stress-ripening protein 2
c82400	-1.37651	down	abscisic acid inducible protein TAS14-like
**ET related genes**			
c36806	1.916629	up	ethylene-responsive transcription factor 2-like
c81731	1.689691	up	ethylene-responsive transcription factor RAP2
c73789	-2.56806	down	1-aminocyclopropane-1-carboxylate oxidase 1
c45524	-1.26547	down	1-aminocyclopropane-1-carboxylate oxidase 1
c59922	-1.1586	down	adenylate isopentenyltransferase 5
c72189	-4.50739	down	ethylene-responsive transcription factor ABR1
c68758	-1.70653	down	ethylene-responsive transcription factor 1B-like
c33523	-1.41461	down	Ethylene-responsive transcription factor 2
c69748	-1.12601	down	Ethylene-responsive transcription factor 1
c64904	-1.0153	down	ethylene response factor,
**SA related genes**			
c83203	1.224551	up	syntaxin-121-like
c81170	-1.20669	down	serine hydroxymethyltransferase, mitochondrial-like

**JA related genes**			
c68003	1.086145	up	allene oxide cyclase
c67550	1.013442	up	jasmonate ZIM domain protein a
c81486	1.494959	up	CER6
c86852	-5.65926	down	coronatine-insensitive protein 1-like
c82769	-2.91773	down	jasmonic acid 2
c33700	-1.49625	down	jasmonate ZIM domain protein b
**SL related genes**			
c76015	1.208508	up	carotenoid cleavage dioxygenase 4
